# BnDGAT1s Function Similarly in Oil Deposition and Are Expressed with Uniform Patterns in Tissues of *Brassica napus*

**DOI:** 10.3389/fpls.2017.02205

**Published:** 2017-12-22

**Authors:** Cuizhu Zhao, Huan Li, Wenxue Zhang, Hailan Wang, Aixia Xu, Jianhua Tian, Jitao Zou, David C. Taylor, Meng Zhang

**Affiliations:** ^1^College of Agronomy, Northwest A&F University, Yangling, China; ^2^Research Center of Hybrid Rape, Yangling, China; ^3^National Research Council of Canada, Saskatoon, SK, Canada

**Keywords:** triacylglycerol, acyl-CoA:diacylglycerol acyltransferase 1, expression patterns, duplicated genes, oil accumulation

## Abstract

As an allotetraploid oilcrop, *Brassica napus* contains four duplicated Acyl-CoA:diacylglycerol acyltransferase 1 (*DGAT1*) genes, which catalyze one of the rate-limiting steps in triacylglycerol (TAG) biosynthesis in plants. While all four *BnDGAT1*s have been expressed functionally in yeast, their expression patterns in different germplasms and tissues and also consequent contribution to seed oil accumulation *in planta* remain to be elucidated. In this study, the coding regions of the four *BnDGAT1s* were expressed in an Arabidopsis *dgat1* mutant. All four BnDGAT1s showed similar effects on oil content and fatty acid composition, a result which is different from that observed in previous studies of their expression in yeast. Expression patterns of *BnDGAT1s* were analyzed in developing seeds of 34 *B. napus* inbred lines and in different tissues of 14 lines. Different expression patterns were observed for the four *BnDGAT1*s, which suggests that they express independently or randomly in different germplasm sources. Higher expression of *BnDGAT1s* was correlated with higher seed oil content lines. Tissue-specific analyses showed that the *BnDGAT1*s were expressed in a uniform pattern in different tissues. Our results suggest that it is important to maintain expression of the four *BnDGAT1s* for maximum return on oil content.

## Introduction

As an oil crop with global production exceeding 60 million tons (Bancroft et al., 2011), *Brassica napus*, known as oilseed rape, is an allotetraploid (AACC) species that contains two sets of homologous chromosomes originating from its diploid progenitors, *Brassica rapa* (AA) and *Brassica oleracea* (CC), which underwent a triplication event (Nagaharu, 1935; Lysak et al., 2005; Paterson et al., 2006; Chalhoub et al., 2014). Genome duplication and natural hybridization led to gene redundancy in *B. napus*. The genome of *B. napus* is much more complex and divergent than that of *A. thaliana* (Lukens et al., 2003; Town et al., 2006). Most of the multiple gene copies in *B. napus* occur as homologous pairs, one from the A-genome and one from the C-genome (Parkin et al., 2003).

Duplicated genes commonly exist in other important crops such as wheat (*Triticum aestivum*) and cotton (*Gossypium* L.), and are usually caused by polyploidization (Otto, 2007; Jiao et al., 2011). Gene redundancy is thought to allow plants to adapt to varying environments (Comai, 2005). Homologous genes show divergence of function and expression in many plants. *B. napus* has three *GPAT4* (*sn*-1-glycerol-3-phosphate acyltransferase 4) homologs (*BnGPAT4s*) and these exhibit functional divergence in both expression pattern and activity (Chen et al., 2011). Three *WLHS1* (wheat LEAFY HULL STERILE1) homoeologous genes have evolved into functionally divergent forms and have different effects on flowering time in wheat (Shitsukawa et al., 2007). Alterations in gene expression upon polyploidization have been reported in many plant species, including Arabidopsis, cotton, wheat, *Senecio*, *Brassica*, and even in recent early generations of the natural allopolyploid, *Tragopogon mirus* (Flagel et al., 2008; Chaudhary et al., 2009; Buggs et al., 2010; Liu et al., 2011). To overcome genome instability, polyploid species tend to acquire diploid-like characteristics through massive gene loss, genomic reorganization through silencing, and unequal expression of homologous genes (Adams et al., 2003; Chen et al., 2007). In barley, the two copies of a germin-like gene were expressed unequally in different tissues; one copy was predominantly expressed in developing shoots, while another copy was expressed in the pericarp, spikelets, and seedling roots (Federico et al., 2006). In allopolyploid cotton, the expression of two *alcohol dehydrogenase AdhA* homoeologs varied in response to cold, dark, or submergence stress (Liu and Adams, 2007). Similar expression changes were also found in *peroxidase*, *FLC* and *FRUITFULL* genes in *B. napus* (Pires et al., 2004; Zhao et al., 2009; Peng et al., 2015, respectively). Gene silencing of some homoeologs is also common in polyploids, occurring in wheat, cotton and *B. napus* (Adams, 2007; Gaeta et al., 2007). However, there have been few studies as to whether the expression pattern of homologous genes is consistent among different cultivars.

Acyl-CoA:diacylglycerol acyltransferase1 (DGAT1; EC 2.3.1.20) is an endoplasmic reticulum (ER)-bound enzyme which catalyzes the acyl-Coenzyme A (CoA)-dependent acylation of *sn*-1,2-diacylglycerol (DAG) to produce triacylglycerol (TAG) (Routaboul et al., 1999; Zou et al., 1999; Jako et al., 2001). DGAT1s are *O*-acyltransferases and are predicted to have between 6 and 12 transmembrane domains and a typical mass of about 60 kDa (McFie et al., 2010). As the final enzyme in TAG biosynthesis, DGAT1 is critical to oilseed development and has been highlighted as a genetic engineering target to increase storage lipid production in plants and microorganisms. Lacking function of DGAT1 in the *A. thaliana* *dgat1* mutant, AS11, leads to a 30% decrease on seed oil content, and a strongly increased proportion of 18:3 and reduction in 20:1 in TAGs, compared to wild type (Katavic et al., 1995). Interestingly, in the DGAT1 mutant, enhanced phosphatidylcholine:diacyglycerol transacylase (PDAT) activity is thought to compensate for lack of DGAT1 catalysis, to maintain significant oil content for viability (Taylor, unpublished). Additionally, DGAT1 plays important roles in organ development (Lock et al., 2009; Zhang et al., 2009). Homologous genes of *DGAT1* have been identified/annotated in more than 50 organisms and in many plant species, where they have been shown to play an important role in seed oil accumulation. There have been numerous studies in both plants and microorganisms where over-expression of DGAT1s has led to increased TAG content (Kamisaka et al., 2007; Taylor et al., 2009; Andrianov et al., 2010; Tai and Stephanopoulos, 2013; Yu et al., 2013).

In *B. napus*, several cDNA sequences of putative DGAT1s have been isolated (GenBank ID: AF164434.1 by Nykiforuk et al., 1999; GenBank ID: AF155224.1 by Nykiforuk et al., 1999; GenBank ID: AF251794.1, by Brown et al., 2000, unpublished). All four duplicated genes of *BnDGAT1* including AF164434.1, were annotated by our group (Li et al., 2014). BnDGAT1 and BnDGAT2 (later correctly renamed as BnDGAT1-1) were transiently induced in cell suspension cultures by higher sucrose concentration and function assessed using both transcript expression and *in vitro* DGAT activity assays (Nykiforuk et al., 2002). Overexpressing BnDGAT1 in *B. napus* increased the DGAT1 activity and also increased oil content under field drought conditions (Weselake et al., 2008). A purified recombinant BnDGAT1 (BnaC.DGAT1.a) enzyme showed a distinct substrate preference for α-linolenoyl-CoA (Caldo et al., 2015). The coding regions of the four *BnDGAT1s* were over-expressed in *Saccharomyces cerevisiae* and were found to increase TAG accumulation; the homoeologs showed significant differences in rates of TAG synthesis (Aznar-Moreno et al., 2015; Greer et al., 2015). Transcriptional analysis by RNA-seq showed that BnDGAT1-1 and BnDGAT1-2 were highly expressed, while BnDGAT1-3 and BnDGAT1-4 were lower in expression (Aznar-Moreno et al., 2015). In this study, we individually expressed the four *BnDGAT1s* in the *A. thaliana* *dgat1* mutant, AS11, to test their function *in planta*. Thirty-four cultivars or inbred lines (hereafter referred to as “lines”) of *B. napus* were selected to analyze the four *BnDGAT1* expression patterns and their relationship to seed oil content. Tissue-specific expression of *BnDGAT1*s was also analyzed in 12 lines.

## Materials and Methods

### Plant Materials

The Arabidopsis *dgat1* knock-out mutant, AS11, purchased from NASC (CS3861), was used as the host for BnDGAT1 functional analysis experiments and the same genetic background Col-0 wild type was used as a control. All *A. thaliana* plants in this study were grown on a soil mixture (PINDASTRUP PLUS PEAT substrate: vermiculite: pearlite = 3: 1 : 1, Pindstrup Mosebrug A/S, Fabriksvej 2, 8550 Ryomgaard, Denmark) in a growth room at 22°C, and a 16 h/8 h light/dark cycle at a fluorescent light intensity of 100 μmol m^-2^ s^-1^.

The expression patterns of *BnDGAT1* homologs were examined in different germplasm sources. In order to investigate the relationship between oil content and BnDGAT1 expression patterns, 14 lines with high seed oil contents (>50%) and 20 lines with lower seed oil contents (<45%) were selected. According to our breeding records, these lines showed relatively stable seed oil contents after more than 5 years of breeding. Because of long-term breeding, there are a few lines with extremely low oil content in our germplasm collection. Line designations are listed in Supplementary Table S1. In this study, all lines were grown and sampled in open fields.

Twenty-eight DAP (days after pollination) developing seeds from all thirty-four *B. napus* lines and 21 DAP developing seeds from 11 *B. napus* lines, were sampled for RNA extraction (Perry and Harwood, 1993). Tissue samples of roots, stems, rosette leaves, and the third cauline leaves of four *B. napus* lines (94-228, 1721-1B, 1941 and Danza 875) were collected at 28DAP and additionally, sepals, petals, stamens and pistils were harvested from these four lines at flowering, for expression analyses of BnDGAT1s. Based on our initial results of BnDGAT1s expression profiling in developing seeds, 12 lines with various BnDGAT1s expression patterns were selected for further analysis. Rosette leaves and flowers of these 12 lines were sampled for tissue-specific expression. Mature seeds from all these lines were collected for measurements of oil content and fatty acid composition of TAGs.

### Sequence Analysis

DGAT1 protein sequences were acquired from GenBank according to the annotation of sequences similar to BnDGAT1s. A Neighbor-joining (NJ) tree was constructed by MEGA6.0 (Center for Evolutionary Medicine and Informatics, Tempe, AZ, United States). DGAT1 protein sequences of the four BnDGAT1s were aligned by DNAMAN.

### Heterologous Expression in AS11

The coding region of four duplicated *BnDGAT1s* were cloned into plant expression vector pK7WG2D (Invitrogen) and construct integrity was confirmed by sequencing. PCR primers are listed in Supplementary Table S2. The vectors were transformed into *Agrobacterium tumefaciens* strain GV3101 and then transformed into *A. thaliana dgat1* mutant AS11. Plant transformation was performed using the floral dip method (Clough and Bent, 1998).

### Homoeolog-Specific PCR Primer Design

Three pairs of primers were designed to detect the expression of *BnDGAT1-1*, *BnDGAT1-2* and the combined expression of *BnDGAT1-3* and *BnDGAT1-4*. The primers are listed in Supplementary Table S2. All primers were verified by amplifying respective plasmids with the four *BnDGAT1s* homeologs (Supplementary Figure S4).

### Semi-quantitative-PCR Analysis of BnDGAT1s

Total RNA was isolated from various plant organs using Trizol reagent following the manufacturer’s instructions (Invitrogen, Carlsbad, CA, United States) and was treated with RNase-free DNaseI (Qiagen, Hilden, Germany). Total RNA (2 μg) was reverse- transcribed into cDNA using the M-MLV RTase cDNA Synthesis Kit (Takara, Shiga, Japan). The cDNAs were then used as templates in 20 μl semi-quantitative-PCR reactions using homeolog-specific PCR primers. Actin was used as the control gene. The semi-quantitative-PCR reactions for *BnDGAT1-1, 2* and *3/4* were carried out under similar conditions (95°C for 30 s; 48–53°C for 1 min; 72°C for 30 s; 29 cycles) with a slight adjustment in annealing temperature (53°C for *BnDGAT1-1*, 53°C for *BnDGAT1-2,* and 48°C for *BnDGAT1-3/4*). Seven microliters of each PCR product was loaded onto a 2% agarose gel for electrophoresis.

### Fatty Acid Analysis of Arabidopsis Seed Oils and Measurement of *B. napus* Seed Oil Content and Acyl Composition

Fatty acid analysis of Arabidopsis seeds followed the method of Li et al. (2006) with minor modifications. Thirty randomly-picked seeds per replicate were used for measuring seed weights after 48 h desiccation. Ten micrograms of seeds per replicate were used for fatty acid analysis. Triheptadecanoin was used as an internal TAG standard. Two milliliters of methanolic H_2_SO_4_ 2.5% (v/v) was added to each sample and heated at 80°C for 120 min. After neutralization and extraction with hexane, the fatty acid methyl ester profile was analyzed by gas chromatography on a DB23 column with flame ionization detection. Fatty acid composition analyses of *B. napus* seeds followed the same protocol except the seeds were dried at 60°C for 3 days and were pulverized before transmethylation.

Oil contents of *B. napus* seeds were measured on an mq20 NMR analyzer, Bruker optics (10 mm sample tube diameter, probe head temperature 40°C, environment temperature 25°C). Scans were repeated four times.

## Results

### BnDGAT1s Show Variable Protein Motifs

**FIGURE 1:**
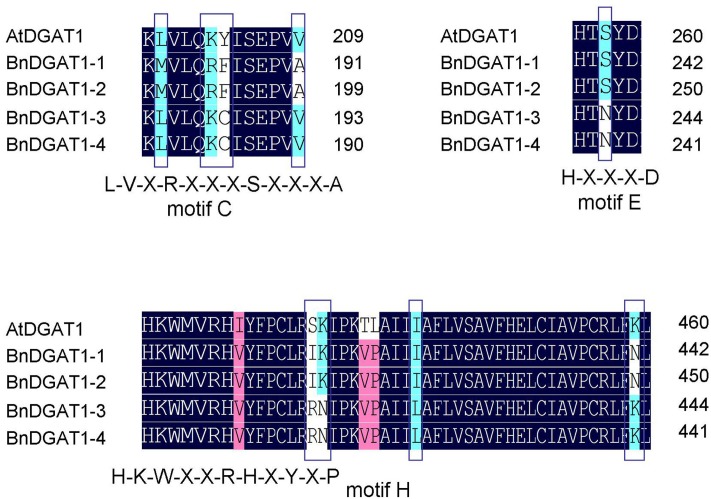
Differentiation of BnDGAT1s on motifs C, E, and H. Sequence alignment of deduced BnDGAT1 and AtDGAT1 amino acid sequences were performed by using DNAMAN. Motifs of an acyl-CoA binding signature spanning active site catalytic residues (motif A), a thiolase acyl-enzyme intermediate binding motif (motif B), a typical targeting site of members of the sucrose non-fermenting (SNF)-related protein kinase 1 (SnRK1) family (motif C), a leucine zipper motif (motif D), motifs common to most annotated DGAT1s (motifs E and F), a fatty acid binding protein signature spanning residues (motif G), and a DAG/phorbol ester binding motif (motif H) were identified. Only motifs C, E, and H show differentiations among BnDGAT1s.

In previous studies, the four BnDGAT1s were heterologously expressed in yeast and found to have different acyl-CoA substrate preferences and varying efficiencies in catalyzing TAG biosynthesis (Aznar-Moreno et al., 2015; Greer et al., 2015). Protein sequence alignment comparisons among BnDGAT1s and the *A. thaliana* DGAT1, revealed eight known signature motifs, five of which were completely identical among the four homoeologs (Kaup et al., 2002), including an acyl-CoA binding signature spanning active site catalytic residues (motif A), a thiolase acyl-enzyme intermediate binding motif (motif B), a leucine zipper motif (motif D), a motif unique to DGATs (motif F), and a fatty acid binding protein motif (motif G) (Supplementary Figure S3). Residue differences were found on motifs C, E, and H (**Figure 1**). However, only one of them appeared in a structurally-important motif, on the first residue in motif C. Motif C (L^180^-V^181^-X-R^183^-X-X-X-S^187^-X-X-X-A^191^) is a typical targeting site of members of the sucrose non-fermenting (SNF)-related protein kinase 1 (SnRK1) family, which are involved in the global regulation of carbon metabolism (Halford and Hardie, 1998). Similar SnRK1 targeting motifs were present in LPAATs from coconut (Knutzon et al., 1999) and meadowfoam (Lassner et al., 1995), and one was previously mentioned in the Arabidopsis DGAT1 (Zou et al., 1999). The first residue of motif C is methionine (M) in BnDGAT1-1 and BnDGAT1-2 while it is leucine (L) in BnDGAT1-3 and BnDGAT1-4. Since this residue is of structural importance, this difference might cause a functional divergence among BnDGAT1s. Other residue differences, including the two on motif C and five on motif E (a motif common to most annotated DGAT1s) and motif H (a DAG/phorbol ester binding motif) were variable residues among other DGAT1s as well.

To understand the evolutionary relationship of BnDGAT1s, 64 DGAT1s from 37 species were identified in GenBank based on the annotation of sequences similar to BnDGAT1s. A phylogenetic tree based on these protein sequences was constructed (Supplementary Figure S1). BnDGAT1-1 and BnDGAT1-3 displayed high sequence homology to DGAT1 genes of *B. rapa* (AA genome), while BnDGAT1-2 and BnDGAT1-4 were homologous to DGAT1 genes identified in *B. oleracea* (CC genome). Additionally, the chromosomal locations of the four *BnDGAT1s* were investigated according to genomic sequencing data on-line^1^. However, there are only three *BnDGAT1* genes in this database; one of them shows partial sequence identity to *BnDGAT1-1* and partial sequence identity to *BnDGAT1-2*, suggesting an error in sequence assembly. Only *BnDGAT1-3* could be assigned, located on chromosome A07 (40438–43985 bp) (Supplementary Figure S2).

### Heterologous Expression of All Four BnDGAT1s in Arabidopsis dgat1-1 Fully Restore Oil Content to Wild Type Levels and Partially Complement the Fatty Acid Composition Aberrations

**FIGURE 2:**
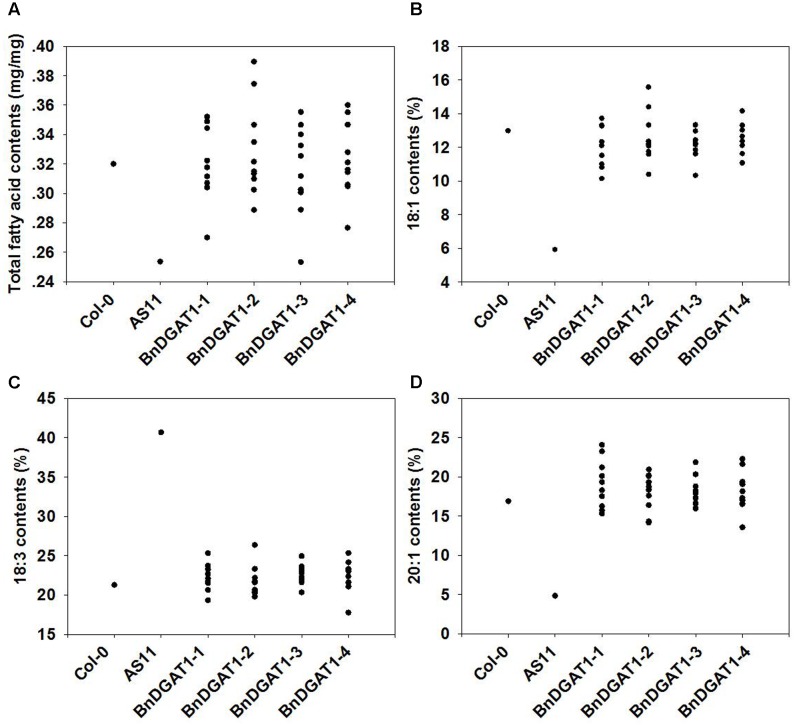
Seed oil contents and fatty acid profiles of BnDGAT1 transgenic lines, AS11 and wild type Arabidopsis. Seed oil contents **(A)**, relative contents of 18:1 **(B)**, relative contents of 18:3 **(C)**, and relative contents of 20:1 **(D)**. Ten independent lines were analyzed for each BnDGAT1 construct. Three biological replicates were analyzed for each line and dots represent their average values.

In order to characterize activities and acyl-CoA substrate preferences of the four *BnDGAT1s* *in planta*, their coding regions were expressed individually in AS11, an Arabidopsis *dgat1* knock out line in the Col-0 genetic background (Katavic et al., 1995). Loss-of-function of DGAT1, as is the case in AS11, leads to decrease of seed oil content and drastically alters its fatty acid profile. To avoid possible complications in interpretation of results due to dosage effects of multiple insertions (Vassileva et al., 2001; Mansur et al., 2005), single-copy transgenic lines were identified by the segregation ratio of T_2_ seeds. Ten of these single insertion lines were randomly selected and characterized; seed oil contents and fatty acid compositions were determined by GC. The results showed that heterologous-expression of any one of the *BnDGAT1s* restored the oil deficiency in AS11 to the oil level found in the wild type (**Figure 2A**). Additionally, all DGAT1s generally complemented the AS11 fatty acid composition changes, restoring them to a composition more like wild type. Three FAs (18:1, 18:3, and 20:1) showed significant reversion to WT proportions in all 40 homozygous *BnDGAT1* AS11 transformants, when compared to AS11. Eicosenoic acid (20:1) increased, and 18:3 decreased to the level of wild type (**Figures 2B–D**). In general, the total proportion of mono-unsaturated fatty acids was restored. These results suggested that all of the four expressed BnDGAT1s had DGAT enzyme activity.

Because all four BnDGAT1s were expressed in the same AS11 background without the interference of endogenous/native AtDGAT1 activity, phenotypes of these transgenic lines, to a large extent, reflect activities of the expressed BnDGAT1s. It is worth noting that the four BnDGAT1 transformant sets did not generally show widely divergent differences in oil accumulation and fatty acid composition, which also suggests that they have similar kinetics and acyl-CoA preferences *in planta*.

### Higher Numbers and Levels of Expression of *BnDGAT1s* Are Correlated with High Oil Germplasm

Although the heterologously expressed cDNAs of *BnDGAT1s* did not show differences in activity (as measured by oil content), there might be differences in gene expression. In a previous study, expression levels of *BnDGAT1-1* and *BnDGAT1-2* were much higher than those of *BnDGAT1-3* and *BnDGAT1-4* in middle- and late-stage embryogenesis (Aznar-Moreno et al., 2015). We were interested in whether this would remain true in other germplasm sources. In other words, are all four DGAT1s crucial to TAG synthesis in *B. napus*? In order to address these questions, characterization of *BnDGAT1s* expression patterns in different germplasms was undertaken. However, the very high sequence similarity/homology of the four genes renders it difficult to discriminate between them by traditional real-time PCR or northern-blot. Although the expression patterns of the four genes in *B. napus* were identified by transcriptome sequencing (Aznar-Moreno et al., 2015), it would be intractable and costly to quantitatively test dozens of accession lines and tissues by RNA-Seq. Therefore, as a compromise we selected semi-quantitative PCR (semi-q PCR) for testing the expression difference of the four *BnDGAT1s*. Even so, *BnDGAT1-3* and *BnDGAT1-4* were still too similar to be distinguishable from each other; thus one pair of semi-q PCR primers was designed to measure the combined expression of *BnDGAT1-3* and *BnDGAT1-4*. The other two pairs of semi-q PCR primers were designed to measure the expression of *BnDGAT1-1* and *BnDGAT1-2*, independently. Semi-q PCR measurements of expression were carried on 28 DAP developing seeds of 34 inbred lines and 21 DAP developing seeds of 11 inbred lines. The results showed that there were different expression patterns in this germplasm collection. Three/four *BnDGAT1s* distinctly expressed in 13 lines and all four genes weakly expressed in two lines. Not all *BnDGAT1s* strongly expressed in the other 19 lines (Supplementary Figure S5). It is worth noting that the expression patterns are identical in the same germplasm at two stages of developing seeds. Taken in the context of the above results of expression in Arabidopsis, and AS11 in particular, all four *BnDGAT1* are likely functionally-expressed in different germplasms.

**FIGURE 3:**
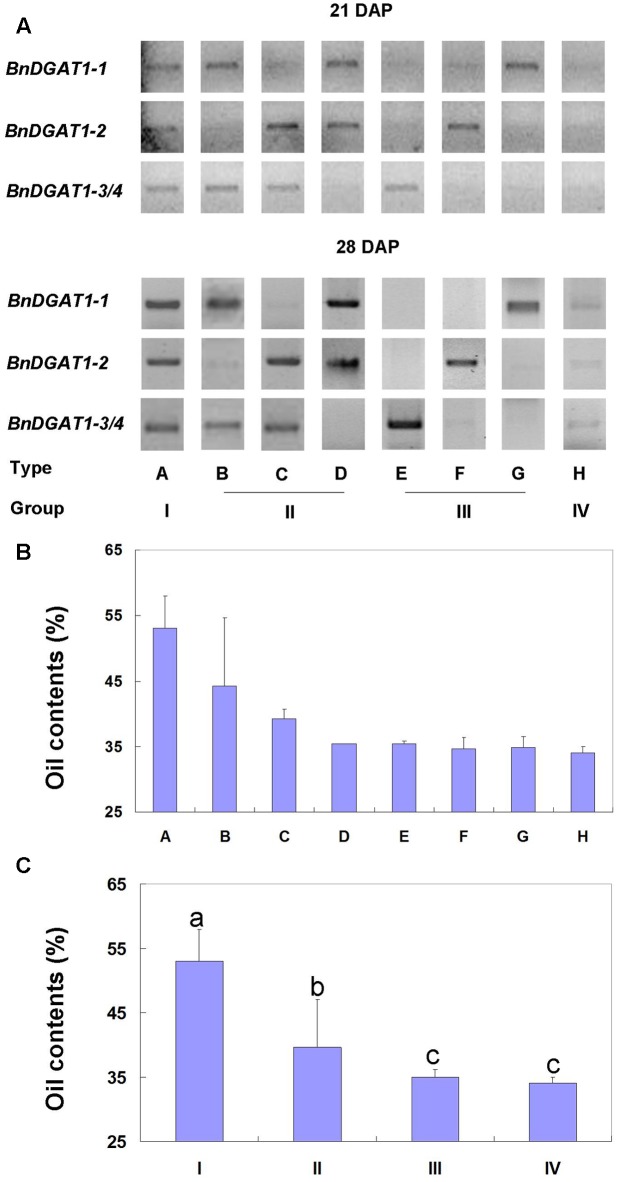
The expression patterns of BnDGAT1s and related seed oil contents in *Brassica napus*. **(A)** Expression patterns of BnDGAT1s in four groups and eight types of *B. napus* lines (each type chose one line as represent). Samples of developing seeds from 21 and 28 DAP stages are from same line. Primers and semi-quantitative PCR conditions are described in Section “Materials and Methods.” **(B)** Seed oil contents of eight types of *B. napus* lines. **(C)** Seed oil contents of four groups of *B. napus* lines. Error bars in B and C indicate SD of oil contents from lines in the types or groups.

Based on strong semi-q PCR band representation of the *BnDGAT1s* identified, there were eight types of expression patterns (**Figure 3A**) and the 34 lines fell into different expression types of *BnDGAT1s* (Supplementary Table S3). The relationship between *BnDGAT1* expression patterns and seed oil contents was studied in all 34 rapeseed lines (Supplementary Table S1). Lines with lower seed oil contents primarily have one or two weaker-expressed or silenced *BnDGAT1s*, examples being lines 1721-1B, QY3, You22 (Supplementary Figure S5); some even have three or four weaker-expressed or silenced *BnDGAT1s*, as in lines in groups III and IV (**Figure 3**). However, this trend was not consistent in all lines. Some lines with the expression of four *BnDGAT1s*, such as QY10 (Supplementary Figure S5), still showed low seed oil content. To further investigate the relationship between seed oil contents and expression patterns of *BnDGAT1s*, average oil contents of each pattern type were calculated. Oil content of type A with three strong semi-q PCR bands was much higher than those of other types (**Figure 3B**). Type B and C showed slightly higher seeds oil contents than the other groups. Considering the similar functions of each DGAT1, lines were grouped by the number of distinct DGAT1 expression bands. There were two sharp bands in types B, C, and D and they were designated as Group II. Group III included types E, F, and G with one sharp band. Group I and Group IV included lines with three sharp or weak bands, respectively (**Figure 3A** and Supplementary Table S3). Average oil contents of groups were calculated and results showed that the higher the expression of *BnDGAT1s*, the higher the oil contents of the groups (**Figure 3C**). These results suggest that seed oil content of the rapeseed lines was closely correlated to the expression level and pattern of *BnDGAT1s*.

BnDGAT1s were reported to show diversity in substrate specificity (Aznar-Moreno et al., 2015). In order to investigate the possible consequence of this difference *in planta*, fatty acid compositions were compared among type G (only *BnDGAT1-1* highly expressed), type F (only *BnDGAT1-2* highly expressed) and type H (none *BnDGAT1s* highly expressed). The proportions of 18:1 were 50–60% in all of these lines and no obvious difference in fatty acid composition was found among these lines (Supplementary Figure S6).

### *BnDGAT1s* Show Similar Expression Patterns in Different Tissues

**FIGURE 4:**
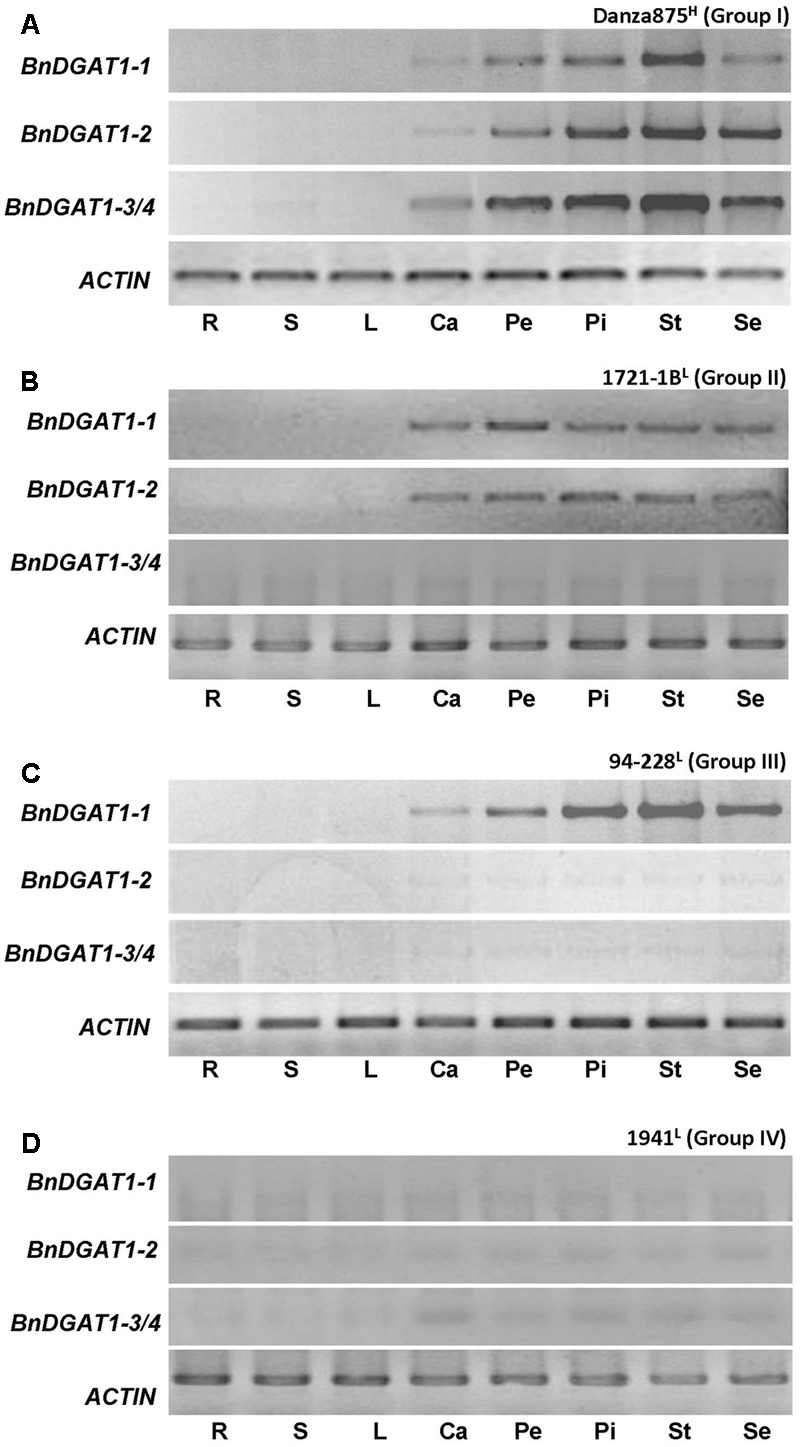
Tissue specific expression patterns of BnDGAT1s in *B. napus*. Roots (R), stems (S), leaves (L) and the third cauline leaves (Ca), of *B. napus* lines from four groups (**A–D**, represent group I–IV respectively) (one line was selected from each group) were collected on 28 DAP (days after pollination) and analyzed by RT-PCR. Petals (Pe), pistils (Pi), stamens (St), and sepals (Se) were collected at same stage and analyzed by RT-PCR. Actin was used as internal reference. Primers and semi-quantitative PCR conditions are described in Section “Materials and Methods.” L indicates low seed oil and H indicates high seed oil.

**FIGURE 5:**
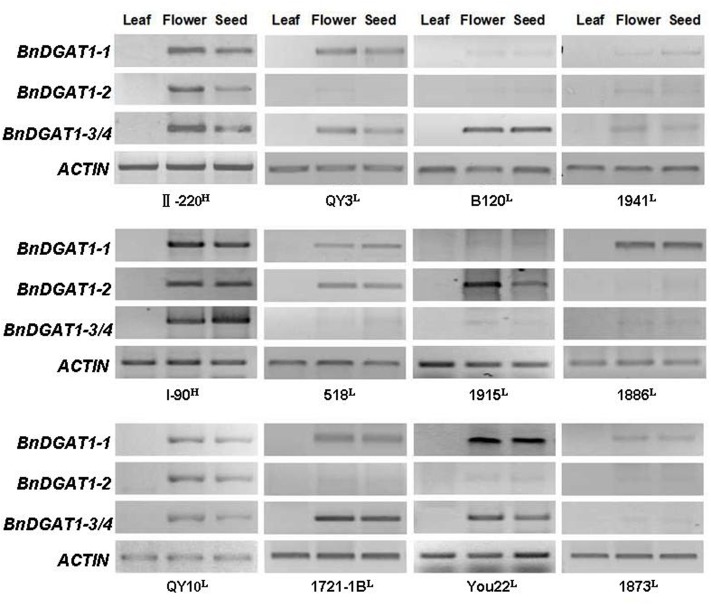
Expression patterns of BnDGAT1s in leaves, flowers, and developing seeds of *B. napus*. Tissue samples were collected on 28 DAP and analyzed by RT-PCR. Actin was used as internal reference. Primers and semi-quantitative PCR conditions are described in Section “Materials and Methods.” L indicates low seed oil and H indicates high seed oil.

To determine whether there are different tissue expression patterns of the four *BnDGAT1s* within the same germplasm background, lines with different *BnDGAT1* expression patterns in developing seeds (Danza875 type A from group I, 1721-1B type B from group II, 94–228 type G from group III and 1941 type H from group IV), were investigated. Roots, stems, the first rosette leaves, the third cauline leaves, sepals, petals, stamens, and pistils and developing seeds (28 DAP) were collected at flowering. Expression analyses of *BnDGAT1*s in these tissues showed that *BnDGAT1*s mainly expressed in reproductive organs. Stamens were the organ with the strongest expression of the *BnDGAT1*s, followed by pistils, 28 DAP seeds and petals. There was no detectable expression of *BnDGAT1*s in roots, stems, and rosette leaves at this stage. However, it was interesting to note that there was weak expression of *BnDGAT1*s in cauline leaves (**Figure 4**). Other than the expression level, the expression patterns of the four *BnDGAT1s* in different organs were identical to that in developing seeds (**Figure 4**). In order to test the similarity of *BnDGAT1* expression pattern in other lines, samples of leaves, flowers, and developing seeds were collected from 12 lines with different *DGAT1* expression patterns. The *BnDGAT1* expression pattern in flowers was identical to that in developing seeds in all 12 lines and no exceptions were found in any of the 12 lines that were tested. *BnDGAT1*s that were not expressed in developing seeds, like *BnDGAT1-2* in QY3, B120, 1886, were also not expressed in other organs (**Figure 5**). These genes may be silenced in these lines. The data suggest that the four *BnDGAT1*s have consistent expression patterns in different tissues, especially in flowers and developing seeds, spanning the generative phase.

## Discussion

Duplication of genes is considered to be a driving force in plant evolution. Duplicated genes may maintain their original function, diverge to acquire neo-functions, or lose function. As the most important oil crop in the Brassicaceae family, *B. napus* is a natural allotetraploid with a short history of only several 1000 years. However, its parental *Brassica* species underwent a whole-genome triplication 5–15 million years ago (Beilstein et al., 2010; Wang et al., 2011). It is necessary to investigate the possible functional divergence of their duplicated genes, especially the oil-related genes. As the most important structural gene whose function is solely dedicated to the biosynthesis of triacylglycerol, the four BnDGAT1s have been shown to be functional when expressed in yeast (Aznar-Moreno et al., 2015; Greer et al., 2015). Overexpressing one of the *BnDGAT1s* resulted in an increased oil content in transgenic *B. napus* lines (Taylor et al., 2009). However, until now, the functions of other duplicated BnDGAT1s have not been tested *in planta*.

In this study, four *BnDGAT1s* were expressed in the Arabidopsis *dgat1* mutant AS11 which does not have a functional DGAT1. All four *BnDGAT1s* complemented the mutant oil deficiency (**Figure 2A**). Because there was no interference from the endogenous DGAT1 in the AS11 background, the DGAT contributions to oil content and composition could be evaluated by the phenotypes of single-insert transgenic lines. The relative proportions of all fatty acids were restored to the levels of wild type (**Figure 2**). These results indicate that all four *BnDGAT1s* were functionally expressed in Arabidopsis. Although positional effects were not ruled out by results from a large number of lines, similar complementation patterns of oil content and fatty acid compositions in tested transgenic lines suggested similar functions of four BnDGAT1s in oil accumulation. Oil contents were similar to each other in the expression types E, F and G, wherein only one expressed band was identified (**Figure 3**). Additionally, two *BnDGAT1-1* expressing lines and two *BnDGAT1-2* expressing lines showed similar fatty acid profiles in seeds (Supplementary Figure S6). Although oil content and fatty acid profile are decided by many factors, our results suggest that the duplicated BnDGAT1s generally function similarly in seed oil accumulation of *B. napus.* However, there are studies suggesting some degree of functional divergence. BnDGAT1-2 and BnDGAT1-3 are highly active in TAG biosynthesis when expressed in yeast, producing about twice the oil of that synthesized by BnDGAT1-1 and BnDGAT1-4 (Aznar-Moreno et al., 2015). In another report, the rates of TAG accumulation were in the order: BnaC.DGAT1.a (BnDGAT1-2) > BnaA.DGAT1.a (BnDGAT1-1) > BnaA.DGAT1.b (BnDGAT1-4) > BnaC.DGAT1.b (BnDGAT1-3) (Greer et al., 2015). These results are different from the current study, which points to the importance of *in planta* expression as compared to what can be inferred from yeast expression studies. Differences were found on three motifs of predicted BnDGAT1 proteins (**Figure 1**); thus, while beyond the scope of this study, we could not rule out the possibility of other diversified neo-functions of the four BnDGAT1s beyond DGAT1 activity.

Expression diversification of duplicated genes happens frequently in polyploidy (Blanc and Wolfe, 2004), even in recent polyploidy (Buggs et al., 2010). *BnDGAT1-1* and *BnDGAT1-2* showed significantly higher expression levels than did *BnDGAT1-3* and *BnDGAT1-4* in one cultivar according to RNA-seq data reported by Aznar-Moreno et al. (2015). It is therefore important to know the expression pattern of *BnDGAT1s* in other *B. napus* germplasm. In the current study, semi-q PCR was used to test expression patterns of *BnDGAT*1 in over 30 accession lines. The high similarity of these four *BnDGAT1s* prevented us from discriminating accurately between *BnDGAT1-3* and *BnDGAT1-4* but we were able to do so effectively with *BnDGAT1-1* and *BnDGAT1-2* (Supplementary Figure S4). It was found that any one, or a combination of the four *BnDGAT1s*, could be expressed in the tested lines (**Figure 3** and Supplementary Figure S5). The expression pattern in a previous report (Aznar-Moreno et al., 2015) fits into just one type of expression pattern delineated in this study, that being expression type D in group II (**Figure 3**). It suggests that these *BnDGAT1s* express randomly or independently in different sources of germplasm. To our knowledge, there are few studies to have examined the expression of duplicated genes in large numbers of germplasm of any species. Our results suggest that it is necessary to identify gene expression of duplicated genes in accessions with different genetic backgrounds in order to interpret phenotypic traits such as seed oil content.

The expression of duplicated genes may also become diversified in different tissues (Pires et al., 2004; Federico et al., 2006; Zhao et al., 2009; Peng et al., 2015). We surveyed the expression of *BnDGAT1s* in eight tissues from four lines with different expression patterns in seeds. It was found that the *BnDGAT1s*, which were highly expressed in developing seeds, also strongly expressed in floral tissues, such as petals, pistils, stamens, and sepals; the same *BnDGAT1s* also moderately expressed in cauline leaves. Very weak or even undetectable expression of all four *BnDGAT1* was observed in roots, stems and leaves. Further detection showed that the expression pattern of *BnDGAT1s* in flowers was identical to that in developing seeds in 12 additional lines (**Figure 5**). These results further suggest that there was no divergence of expression patterns among *BnDGAT1s* in any of the tested tissues and the expression pattern is concordant in different tissues. This consistency will enable *BnDGAT1* expression-related selection by sampling tissues other than developing seeds.

*DGAT1* encodes the final enzyme in triacylglycerol (TAG) synthesis via the Kennedy pathway and its expression and activity is closely related to seed oil content in Arabidopsis (Routaboul et al., 1999; Zou et al., 1999; Jako et al., 2001). In this study, four *BnDGAT1*s exhibited eight different expression types in four different groups (**Figure 3**). When we compared their expression patterns with the resultant seed oil content, a strong correlation became readily apparent. In 20 lines with lower seed oil contents (<45%), 10 lines showed two or more DGAT1s silenced or weakly-expressed, and nine lines showed one DGAT1 silenced or weakly-expressed. All four DGAT1s were highly expressed in 12 of the 14 lines with high oil yield (>50%) (Supplementary Table S3). This close relationship between DGAT1 expression and seed oil content also emerges from typing and grouping the expression patterns vs. respective oil contents (**Figure 3**). PDAT is another contributor in TAG synthesis, by which the *sn*-3 position of DAG is acylated by transfer of an acyl moiety from the *sn*-2 position of PC. It is known to complement the lack of DGAT1 activity in AS11 to allow sufficient TAG for seed development and pollen integrity (Zhang et al., 2009). Therefore, while beyond the scope of the current study, in the future we will examine the relative expression patterns and activities of *B. napus PDAT* homologs in relation to those of the four *B. napus DGAT1* homeologs, to see if they have a co-ordinated contribution to oil content, particularly in cases where there is lower expression of one or more of the *DGAT1* homeologs.

## Conclusion

Our results suggest that, among other genetic markers, it is prudent to select lines with all *BnDGAT1*s expressed when selecting for high oil content in *B. napus* breeding programs.

## Author Contributions

All the authors participated in data analysis and interpretation, and contributed to the writing and editing of the manuscript. MZ and CZ conceived the ideas for the study. CZ and HL contributed to the experimentation.

## Conflict of Interest Statement

The authors declare that the research was conducted in the absence of any commercial or financial relationships that could be construed as a potential conflict of interest.

## Footnotes

1 http://brassicadb.org/brad/
