# Supplementary material for: BnDGAT1s Function Similarly in Oil Deposition and Are Expressed with Uniform Patterns in Tissues of Brassica napus
Source: Front Plant Sci. 2017 Dec 22;8:2205. doi: 10.3389/fpls.2017.02205 (PMC5744481; doi:10.3389/fpls.2017.02205)

Supplementary Material

**BnDGAT1s Function Similarly in Oil Deposition and are Expressed with Uniform Patterns in Tissues of *Brassica napus***

**Cuizhu Zhao, Huan Li, Wenxue Zhang, Hailan Wang, Aixia Xu, Jianhua Tian, Jitao Zou, David Taylor, Meng Zhang***

*** Correspondence:** Meng Zhang: zhangm@nwsuaf.edu.cn

# Supplementary Figures and Tables

## Supplementary Figures


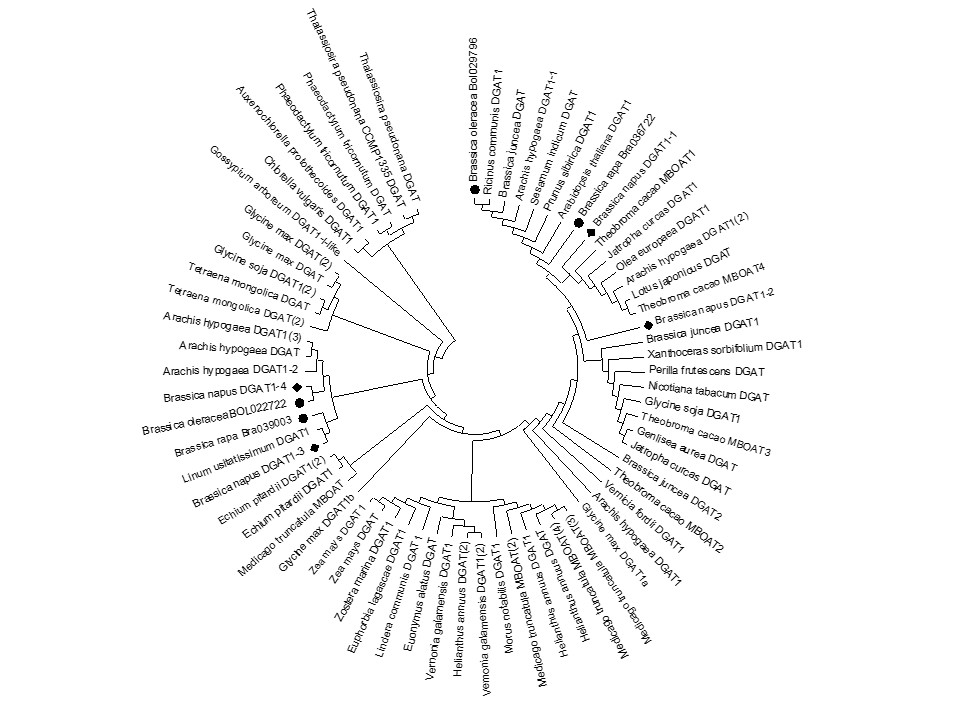


**Supplementary Figure 1.** Neighbour-joining (NJ) tree of 68 DGAT1s from 38 species. DGAT1s of *B. rapa* and *B. oleracea* are indicated with black circles, and *B. napus* DGAT1s are indicated with black diamonds.


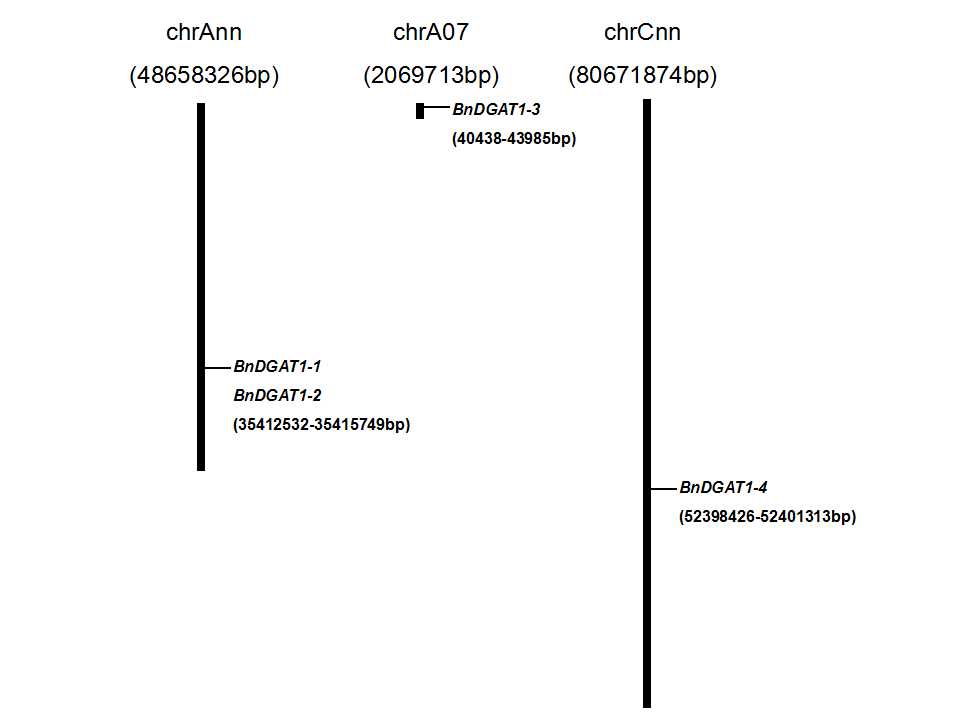


**Supplementary Figure 2.** Locations of BnDGAT1s on *B. napus* chromosome.

**Supplementary Figure 3.** Alignment of deduced BnDGAT1 amino acid sequences. Sequence alignments of BnDGAT1s and AtDGAT1 were performed by using DNAMAN. Motifs of an acyl-CoA binding signature spanning and active site catalytic residues (motif A), a thiolase acyl-enzyme intermediate binding motif (motif B), a typical targeting site of members of the sucrose non-fermenting (SNF)-related protein kinase 1 (SnRK1) family (motif C), a leucine zipper motif (motif D), a DGAT motif (motif E), a DGAT motif (motif F), fatty acid binding protein signature spanning residues (motif G), and a DAG/phorbol ester binding motif (motif H) were highlighted with black bars.

**Supplementary Figure 4.** Specificity test of PCR primers for *BnDGAT1s* homologs. The specificity of primers for BnDGAT1s homologs were tested by using four *BnDGAT1* genes on vector as templates for PCR.


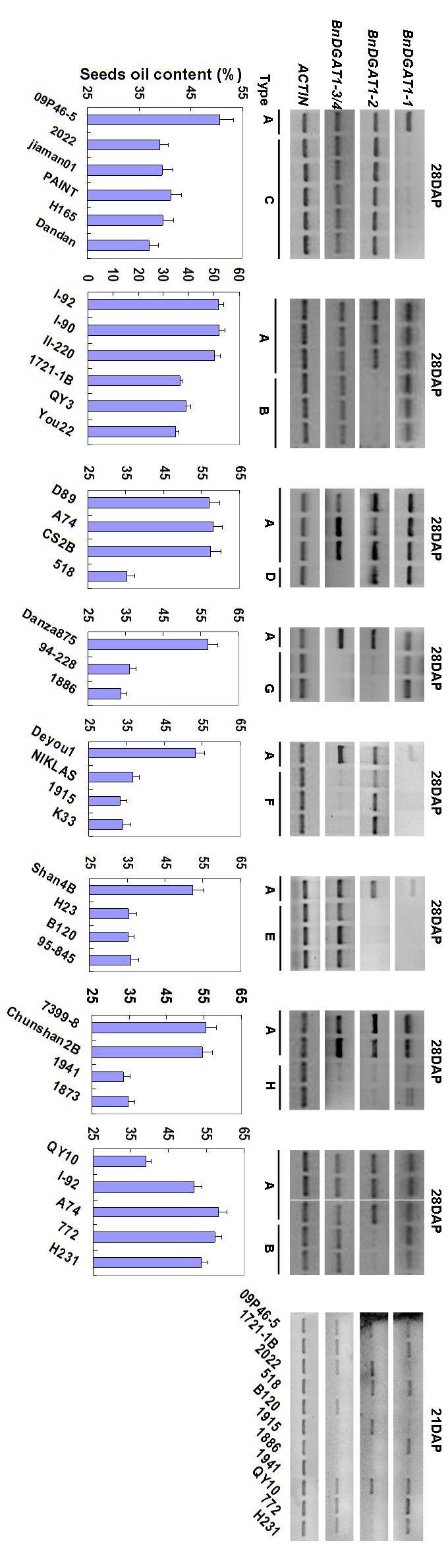


**Supplementary Figure 5.** The expression patterns of *BnDGAT1s* in developing seeds of 34 *B. napus* lines with different seed oil contents. Actin was used as internal reference. Primers and semi-quantitative PCR conditions are described in the Materials and Methods.

**Supplementary Figure 6.** Fatty acid profiles in seeds of 1886, 94-228, NIKLAS, 1941, 1873 and 1915 lines.

## Supplementary Tables

**Table S2 Vector construction and RT-PCR primers**

| **Primers for this research** | |  |
| --- | --- | --- |
| **Full length cDNA PCR primer** | |  |
| BnDGAT1-1/2-FL-F | ATGGCGGTTTTGGATTCTGGA | |
| BnDGAT1-1/2-FL-R | TCAGGACATGGATCCTTTGCGGT | |
| BnDGAT1-3/4-FL-F | ATGGAGA(T/C)TTTGGATTCTGGAG | |
| BnDGAT1-3/4-FL-R | CTATGACATCTTTCCTTTGCGGT | |
| **Homeolog-specific PCR primer** | |  |
| BnDGAT1-1-RT-F | GACGATGTTGGAGCTGCG | |
| BnDGAT1-1-RT-R | AACCTTACATCGCCTCC | |
| BnDGAT1-2-RT-F | GGCCGAAGACGCGA | |
| BnDGAT1-2-RT-R | ATGTAGAACTAAACCAAAAATCAG | |
| BnDGAT1-34-RT-F | TCTTCCTGATTCCGTAACTGT | |
| BnDGAT1-34-RT-R | AGACCTCGGTCATGGTGA | |
| ACTIN-F | GGTGGGGATGGGGCAGAA | |
| ACTIN-R | CCCGCTCGGCAGTGGTG | |


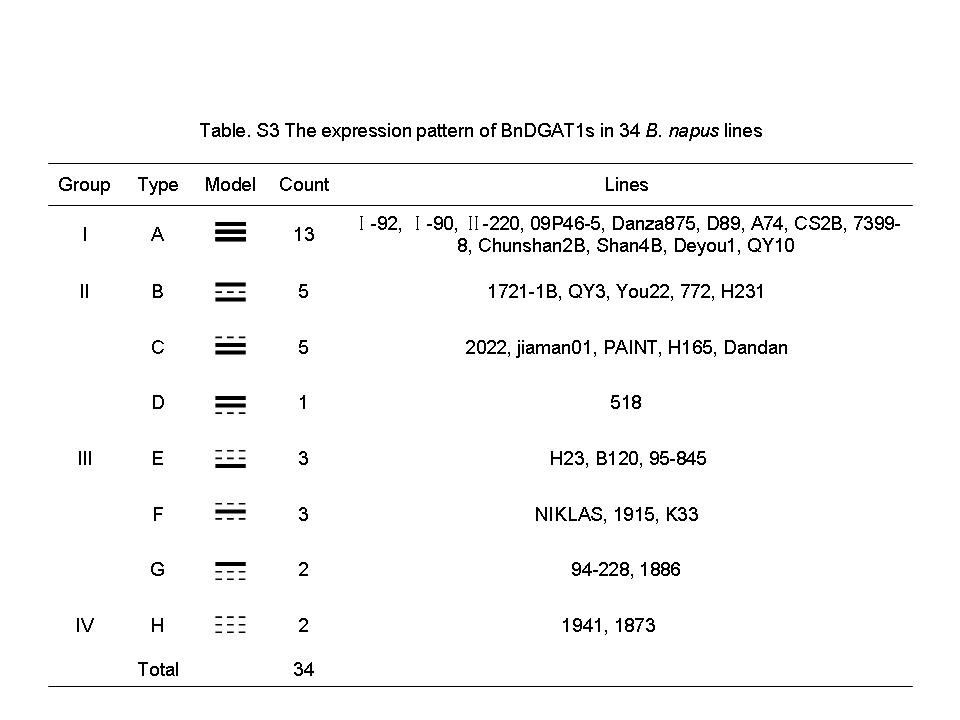

Supplement: Supplementary file 1 [file Data_Sheet_1.DOCX]
